# Dynamic evolution of inverted repeats in Euglenophyta plastid genomes

**DOI:** 10.1038/s41598-018-34457-w

**Published:** 2018-10-30

**Authors:** Anna Karnkowska, Matthew S. Bennett, Richard E. Triemer

**Affiliations:** 1 Department of Molecular Phylogenetics and Evolution, Biological and Chemical Research Centre, Faculty of Biology, University of Warsaw, ul. Żwirki i Wigury 101, 02-089 Warsaw, Poland; 2 Department of Plant Biology, Michigan State University, 612 Wilson Rd, Room# 166 Plant Biology Labs, East Lansing, Michigan 48824 USA

## Abstract

Photosynthetic euglenids (Euglenophyta) are a monophyletic group of unicellular eukaryotes characterized by the presence of plastids, which arose as the result of the secondary endosymbiosis. Many Euglenophyta plastid (pt) genomes have been characterized recently, but they represented mainly one family – Euglenaceae. Here, we report a comparative analysis of plastid genomes from eight representatives of the family Phacaceae. Newly sequenced plastid genomes share a number of features including synteny and gene content, except for genes *mat2* and *mat5* encoding maturases. The observed diversity of intron number and presence/absence of maturases corroborated previously suggested correlation between the number of maturases in the pt genome and intron proliferation. Surprisingly, pt genomes of taxa belonging to *Discoplastis* and *Lepocinclis* encode two inverted repeat (IR) regions containing the rDNA operon, which are absent from the Euglenaceae. By mapping the presence/absence of IR region on the obtained phylogenomic tree, we reconstructed the most probable events in the evolution of IRs in the Euglenophyta. Our study highlights the dynamic nature of the Euglenophyta plastid genome, in particular with regards to the IR regions that underwent losses repeatedly.

## Introduction

Plastids derived from a single endosymbiotic event between a cyanobacterium and the common ancestor of the green algae (including land plants), red algae and glaucophytes - an event called primary endosymbiosis^1^. The plastids of both green algae and red algae were subsequently transferred to other eukaryotic lineages, which gave rise to secondary plastids. Green algal plastids were taken up by two groups of microbial eukaryotes (protists): euglenophytes and chlorarachniophytes^1^. Plastids from red algae were inherited to several algal lineages such as ochrophytes, haptophytes, and others. Although the origin and evolutionary history of plastids in various eukaryotic lineages are very different, almost all plastids contain their own genomes, which share some common features. Most sequenced plastid genomes are from land plants and green algae, but the number of other plastid genomes has increased substantially over the last few years. The vast majority of plastids have a similar structure, genes are dispersed among the inverted repeats (IR) and large and small single-copy (LSC and SSC)^2,3^ regions. The IR regions that contain genes for rRNAs and a variable number of tRNAs and proteins are broadly distributed in all primary and secondary plastids as well as in cyanobacterial genomes^3^. The IR has been suggested to play a role in the replication initiation^4^, genome stabilization^5^, and gene conservation^5,6^. Gene content varies greatly when we consider all photosynthetic eukaryotes, but within each of the evolutionary lineages, gene content and order are conserved features. In some plastid genomes, most notably those of euglenids and land plants, there are also self-splicing introns, which are thought to have invaded the plastid genome on multiple occasions^7,8^. Plastid-encoded group I introns, found in rRNA, tRNA or protein-coding genes, have been reported in apicomplexans, glaucophytes, stramenopiles and Viridiplantae^3^. Group II introns are found in the plastids of cryptophytes, euglenids, and Viridiplantae and usually exist within tRNA or protein-coding genes^3^. The most extreme examples of the group II intron derivatives are characteristic for plastid genomes of euglenids. These include “group III” introns reduced to 73–119 nucleotides, and “twintrons” which are group II introns nested within another group II intron^9–11^. Secondary origin of euglenid plastids and discovery of their unusual introns in plastid genomes triggered further studies on their plastid genomes evolution.

Photosynthetic euglenids (Euglenophyta) constitute a single subclade within euglenids. Their plastids, enclosed by three membranes, arose as the result of the secondary endosymbiosis between phagotrophic eukaryovorous euglenid and the *Pyramimonas*-related green alga^12^. Within photosynthetic euglenids, three evolutionary lineages are distinguished. A single mixotrophic species *Rapaza viridis* forms the most basal lineage^13^. Other photosynthetic euglenids are split into two groups: predominantly marine Eutreptiales and freshwater Euglenales. Euglenales are divided into two families: Phacaceae and Euglenaceae^14,15^. Genomic features of the secondary plastid of euglenids have been studied very intensively in the recent years as more plastid genomes have been sequenced. The first plastid genome of *Euglena gracilis* was sequenced more than two decades ago^16^ but the number of plastid genome sequences rapidly increased since 2012 resulting in 18 euglenid plastid genomes sequenced so far. Three Eutreptiales plastid genomes have been characterized^12,17,18^ and 15 from the Euglenales, including 14 of Euglenaceae^19^ and only one from Phacaceae^20^. The euglenid plastid genome has undergone dynamic changes, including genome reduction due to the gene loss or transfer to the nucleus^12^, the proliferation of introns^11,21^, and genome rearrangements. Intron proliferation has been proposed to be correlated with the number of maturases in the pt genome^20^. The majority of genes among previously sequenced euglenid pt genomes have had the same basic complement of protein-coding genes. However, there have been significant changes in the gene arrangement. More closely related taxa tend to have greater synteny^21^ than more divergent organisms, for which extensive rearrangement have been shown^22^.

Phacaceae comprises three monophyletic genera: *Phacus*, *Lepocinclis*, and *Discoplastis*. The family Phacaceae has been proposed quite recently based on phylogenetic relationships^14,15,23^ and morphological synapomorphy – the presence of numerous, small plastids without pyrenoids. The genus *Phacus* was erected in mid-XIX century^24^ to accommodate taxa from the genus *Euglena* that were rigid and did not undergo metaboly. The genus *Lepocinclis* was established a few years later by Perty^25^ to incorporate taxa from the genus *Phacus* that were not flattened. With the advent of molecular sequencing, the division of taxa between *Phacus* and *Lepocinclis* have been validated and a new genus *Discoplastis* was erected to accommodate several species formerly belonging to the genus *Euglena* and characterized by numerous small plastids without pyrenoids and strong metaboly of the cell^26^. Genus *Phacus* and *Lepocinclis* are species-rich and have been intensively studied on the species-level in the last decade, and several new species have been described^27–31^. In contrast, genus *Discoplastis* comprises only two species^26^.

Here, we report the structural features of the eight newly sequenced plastid genomes of the representatives of the family Phacaceae, including five taxa from *Lepocinclis*, two taxa from *Phacus*, and one *Discoplastis* taxon. We sought to identify the main genomic changes that occurred in the investigated lineages. The examined genomes display considerable variability at all levels except gene content. We also present the phylogenomic trees inferred from the plastid genome sequences. Our results highlight the repeated losses of IR region during the evolution of Euglenophyta.

## Results and Discussion

We investigated eight taxa representing all three genera encompassed in the family Phacaceae and found that the plastid genome experienced important alterations at the level of genome organization and intron prevalence during the evolution of the Phacaceae. Before our comparative analyses, relatively little was known about the extent of chloroplast genomic changes throughout Phacaceae and only one species, *Phacus orbicularis*, has been sequenced^20^. When compared to the characteristics of Euglenaceae pt genomes^19,21,32^, they were very similar in their gene content. However, a few physical traits present within the Phacaceae were remarkable when compared to the rest of the Euglenales and the Euglenophyta.

### General features

**Figure 1:**
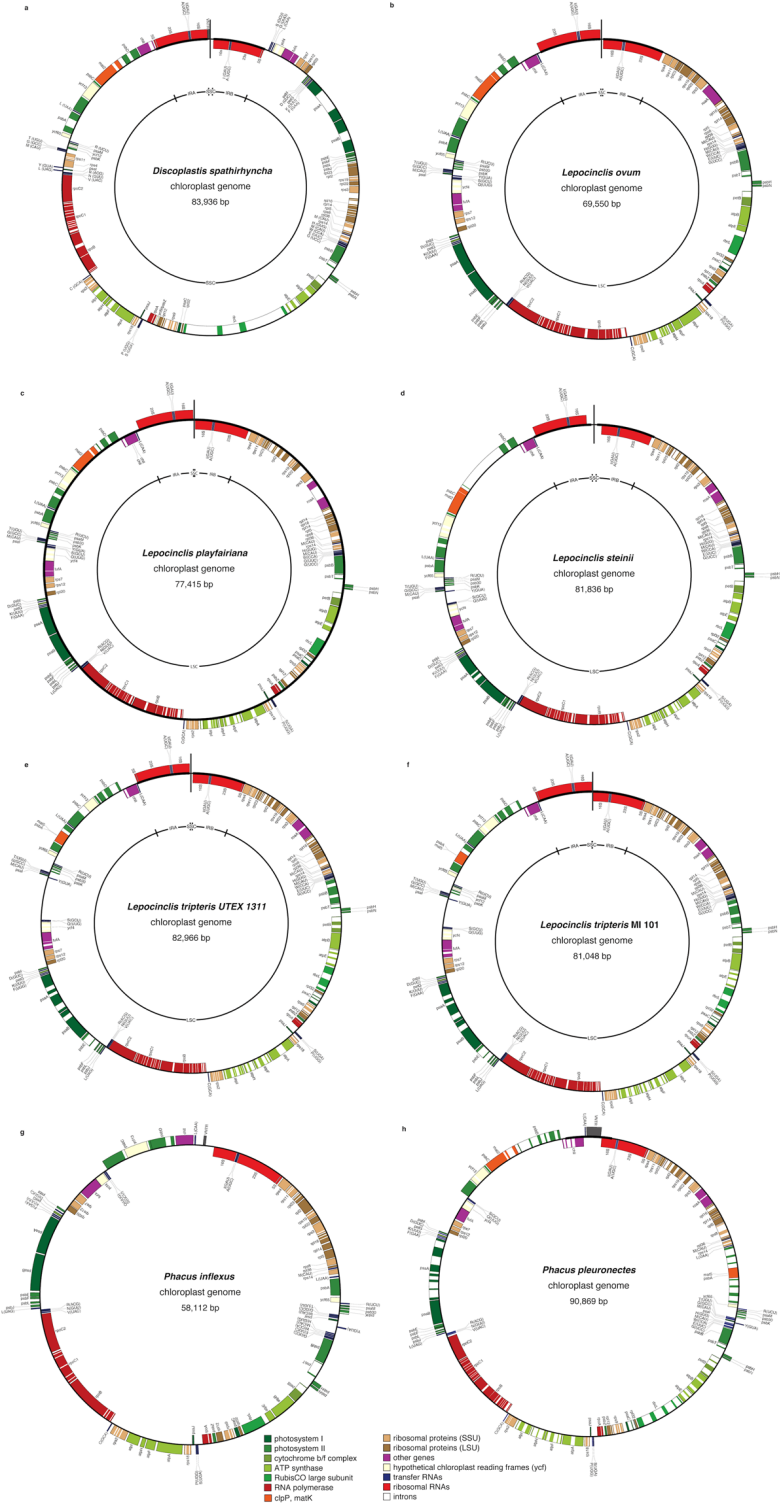
Gene maps of plastid genomes. Boxes of different colors represent genes of similar functional groups. Genes on the outside of the circle are considered on the positive strand, genes inside the circle on the negative strand. (**a**) Plastid genome map of *D. spathirhyncha*, (**b**) *L. ovum*, (**c**) *L. playfairiana*, (**d**) *L. steinii*, (**e**) *L. tripteris* MI 101, (**f**) *L. tripteris* UTEX 1311, (**g**) *P. inflexus*, (**h**) *P. pleuronectes*. The vertical line at the top of the circle indicates that there is a gap and the genomes are not circularized.

Previously, the pt genome of *Eutreptia viridis* contained the smallest genome among Euglenophyta at 65,513 bp^17^, followed by that of the fellow member of Eutreptiales – *Eutreptiella gymnastica* (67, 622 bp)^12^, leading to the assumption that there was an expansion in the genome size in the freshwater euglenids (Euglenales). Among members of the family Euglenaceae, *Monomorphina aenigmatica*^11^ was identified to contain both the smallest genome (74,746 bp) and the smallest number of introns (53 + 1 unidentified ORF). Sequencing of the first representative of the Phacaceae family – *Phacus orbicularis*^20^ revealed that its pt genome (66, 418 bp) is smaller than any of the known Euglenaceae genomes and comparable in size with the pt genomes of *Eutreptiella gymnastica*. All the strains sequenced in this study possess relatively small pt genomes (below 91 Kb) (Fig. 1, Table 1, Supplementary Table 1) and the *P. inflexus* pt genome (~58 Kb) was identified as the smallest Euglenophyceae pt genome sequenced to date, even smaller than the genome of *Eutreptiella gymnastica*. That might suggest that the pt genome of *P. inflexus* underwent reduction, which could be explained by the loss of *mat2* and *mat5* and subsequently a smaller number of introns, along with the low average length of genes (531 bp) (Table 1), lower than those of *Eutreptia viridis* (587 bp).

**Table 1:** Physical characteristics of Phacaceae pt genomes. New pt genomes in Bold. PCG = Protein-Coding Gene(s); Avg. = Average, ≥ = at least. Intron space included when calculating average protein-coding gene length. *P. orbicularis* data from Kasiborski *et al*.^20^.

Taxon	Accession	Size (bp)	IR	G + C (%)	PCG	Avg. PCG length (bp)	tRNAs	rRNAs	Introns (no.)	Avg. no of introns/PCG	PCG with introns	
											no.	%
Discoplastis spathirhyncha	MH898670	≥83,936	2	29.3	61	1,126.6	29	6	59	0.97	31	50.8
Lepocinclis ovum	MH898674	≥69,550	2	30.3	62	884.4	28	4	50	0.81	23	37.1
Lepocinclis playfairiana	MH898671	≥77,415	2	31.9	62	993.5	29	4	79	1.27	33	53.2
Lepocinclis steinii	MH898672	≥81,836	2	22.7	63	1,085.0	29	6	81	1.29	35	55.5
Lepocinclis tripteris (MI)	MH898668	≥81,048	2	27.3	62	1,013.6	29	6	94	1.52	39	62.9
Lepocinclis tripteris (UTEX)	MH898669	≥82,966	2	27.4	62	1,014.6	29	6	95	1.53	39	62.9
Phacus inflexus	MH898667	58,112	1	29.4	60	531.3	27	3	29	0.48	16	26.7
*Phacus orbicularis*	KR921747	≥66,418	1	27.2	61	872.9	27	3	67	1.10	34	55.7
Phacus pleuronectes	MH898673	90,869	1	24.6	63	1,295.6	27	3	104	1.65	38	60.3

### Phylogenomic analyses

**Figure 2:**
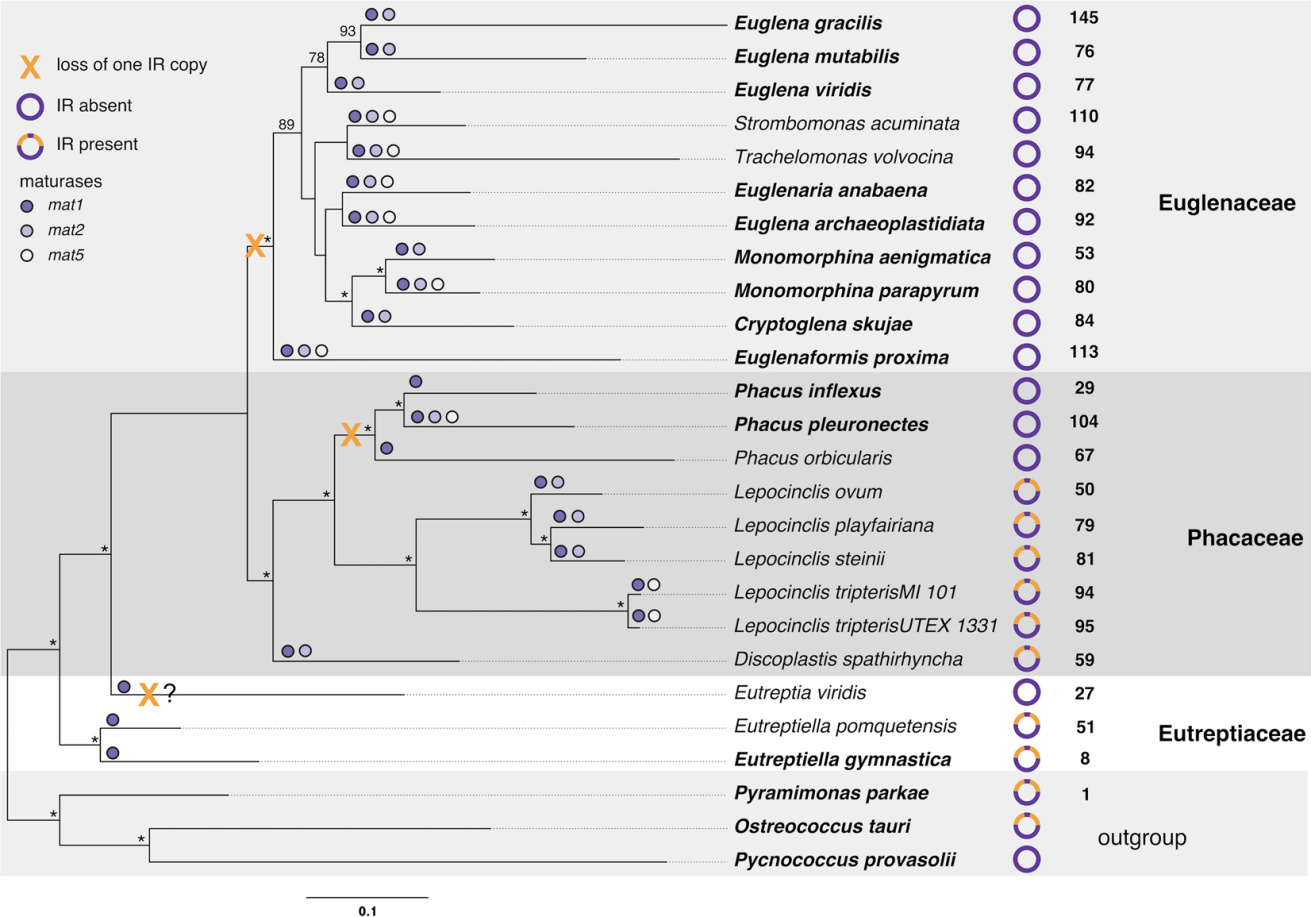
Phylogenetic relationship among the Euglenophyta, with number of introns, presence of maturases and inverted repeat (IR) losses, indicated. The best-scoring maximum likelihood (ML) tree inferred from 57 cpDNA-encoded proteins and two rRNA genes is presented. The numbers on the nodes indicate the ML bootstrap support (bs). An asterisks (*) indicates that the corresponding branch received a BS value of >95%; values below <70% are not presented. The scale bar denotes the estimated number of amino acid substitutions per site. The plastid structure and number of introns are depicted next to the name of the taxa. Number and type (*mat1*, *mat2*, *mat5*) of maturases are denoted on the branches by dots. Species names in bold indicate that their plastid genome sequence is complete.

Before comparing the gene content and gene organization of the examined genomes, here we present the phylogenetic context required to interpret those results. Our chloroplast phylogenomic analyses were carried out using amino acid and nucleotide data sets that included representatives of all genera of Euglenophyta (23 taxa in total). The amino acid data set was generated using 57 protein-coding genes (11,499 sites), whereas the nucleotide data set contained two rRNA genes (3,959 sites). The obtained phylogenetic tree (Fig. 2) confirmed sister relationship of Phacaceae and Euglenaceae^15,23,33^. The inferred relationships among genera of Euglenaceae are not well supported, and only those between closely related taxa are in accordance with multigene analyses on nuclear-encoded genes^14,15^. In contrast relationships among Phacaceae species were well resolved and highly supported. *Phacus* and *Lepocinclis* form sister clades with *Discoplastis* represented by *D. spathirhyncha* branching off first. That topology was previously recovered in phylogenetic analyses based on nuclear-encoded genes^14,15^.

### IR presence/absence

The most prominent structural difference observed among analyzed pt genomes is the presence/absence of inverted repeat (IR) regions encoding the rRNA operon. Two identical IR copies containing all five genes (5S, 16S, 23S, *trn*I, *trn*A) making up the standard rRNA operon were present in the pt genome of *Discoplastis*, and all analyzed *Lepocinclis* (except the 5S gene in *L. playfairiana, L. ovum*, and *L. steinii*) but absent from the representatives of *Phacus*. Initial assemblies of the plastid genomes of *Discoplastis* and *Lepocinclis* were not circularized and the resulting contigs differed in the coverage, suggesting duplication. The contigs were joined via PCR reactions and additional PCR reactions were used to extend the pt genome to the reported genomic sequences. The resulting assembly confirmed that the circularization was impossible due to the presence of an inverted repeat of the ribosomal operon. The arrangement of the plastid genomes in *Discoplastis* and *Lepocinclis* was very similar, IRs are separated by a very short SSC, which doesn’t contain any genes, only tandem repeats.

The plastid genomes of land plants and green algae often contain two copies of an IR encoding the rRNA operon^34^, among them Pyramimonas, a prasinophyte alga most closely related to the ancestor of the plastid of Euglenophyta^12,35^. Among previously sequenced pt genomes of euglenids the quadripartite arrangement has been not shown for Euglenaceae, *Phacus orbicularis*, and *Eutreptia viridis* (Eutreptiales). Only the operons of two species of Eutreptiales, *Eutreptiella gymnastica*^12^, and *Etl. pomquetensis*^18^, resemble the operons and their strand orientation in prasinophytes^18,35,36^, whereas one of the copies was most probably lost during the divergence of Euglenales. Although all known pt genomes of Euglenales lack one copy of IR, in *E. gracilis* and *Stromobomonas acuminata*^37^ they have been shown to contain tandemly repeated copies of the rRNA operon^16,38^.

By mapping the presence/absence of IR on the obtained phylogenomic tree, we reconstructed the most probable events in the evolution of IRs in the Euglenophyta (Fig. 2). Our results suggest that the IR regions were most likely lost three times in the course of evolution of the Euglenophyta (if we assume that the plastid genome acquired from a prasinophyte alga was characterized by the typical quadripartite arrangement of the plastid genome). The first possible loss occurred in the genus *Eutreptia*, however we have to take into account that only one representative of that genus was analyzed and its sequence is incomplete^17^. The second loss occurred during the evolution of the genus *Phacus*, as none of the three analyzed species carried two copies of IR. Finally, the third loss most probably appeared in the common ancestor of all taxa belonging to the family Euglenaceae, which were lacking the quadripartite arrangement of the plastid genome. That scenario is more likely than the creation of an IR *de novo* from an IR-less plastid genome^39^. However, some of the Euglenaceae pt genomes are incomplete, and the number of losses might increase with the completion of those genomes. Specifically, pt genome of *Colacium vesiculosum* and *Strombomonas acuminata* have traits of the possible remnant of the IR; they possess an additional piece of 16S rRNA or 23S rRNA, respectively^37^.

The plastid genomes of almost all land plants and algae carry two identical copies of a large IR sequence^40^. It has been shown that the substitution rates are several times lower for the IR relative to the single-copy (SC) regions among several angiosperms^41^ suggesting a major impact of the IR on the rate of plastome sequence evolution. The omnipresence of the quadripartite structure of plastid genome suggest its important role, however loss of the IR regions, although rare, is known from land plants^42^ and several lineages of the green algae, such as prasinophyceans^43^, trebouxiophyceans^40^, and streptophytes^44^. The molecular mechanisms underlying these events remains unclear, although several hypotheses have been proposed based on studies on plants and green algae^39^. It might be a consequence of repeated events of IR contraction, the complete excision of one of the IR sequence or through the differential elimination of the gene sequences from one IR copy. Among the plastid genomes of the Phacaceae, we didn’t observe any intermediate types of IR copies, which might suggest the step-wise loss of one of the IRs. In contrast, in *Etl. gymnastica* two copies of the IR differ, which might indicate that one of them is on its way to be lost^12^. More plastid genomes sequences form the early branching lineages of Euglenophyta is needed to support either of the aforementioned hypotheses.

### Gene content and introns

The rRNA operon was present in all investigated strains and *Discoplastis* and *Lepocinclis* possess two copies. We did not identify any traces of 5S rDNA in two species, namely *L. ovum* and *L. playfairiana*, and only the core of 5S rDNA (27 nucleotides long) in *L. steinii*. The 5S rRNA was also not identified in *Etl. gymnastica* pt genome^12^ and in some green algae, like *Pyramimonas parkeae* and *Pycnococcus provasolii*^35^. However, the absence of 5S rRNA in plastid genomes, in particular among protists, have been previously shown to be the result of difficulties with identification, not the lack of 5S rRNA in those genomes^45^.

The majority of the protein-coding genes and tRNAs were shared among all investigated taxa, except *roaA*, which was absent in *P. inflexus* and *D. spathirhyncha*, and tRNA L-(UAG) which was absent in *L. ovum*. The number of genes encoding proteins and tRNAs was also consistent with other Euglenophyceae taxa^21^. In several taxa, *roaA* (*P. pleuronectes*) and *atpF* (*P. pleuronectes*, *D. spathirhyncha*, *L. steinii*, and *L. playfairiana*) began with alternative start codons, which was also previously observed for other taxa^21^.

We found notable differences among the maturase-encoding genes. The *mat5* gene has patchy distribution along the tree of photosynthetic euglenids, and it was present in two out of seven analyzed species (both strains of *L. tripteris* and *P. pleuronectes*) (Fig. 2). Bennett and Triemer^21^ proposed that *mat5* was gained after the split of Euglenales from the Eutreptiales while identifying three instances in which *mat5* was independently lost within Euglenaceae. However, they also recognized *mat5* in *L. spirogyroides*, which demonstrated that it was gained before the Euglenaceae/Phacaceae split. Kasiborski *et al*.^20^ did not identify *mat5* in the first pt genome of *Phacus* and proposed that this gene was lost in *P. orbicularis* after the split of *Phacus* and *Lepocinclis*. Our analyses of eight additional strains from the Phacaceae rejected the previous hypothesis since in both *Lepocinclis*, and *Phacus mat5* was absent in some taxa but present in others (Fig. 2). It was also absent in *Discoplastis* but based only on one strain we couldn’t draw definite conclusions. Most likely, *mat5* was present in the ancestor of all Euglenales. The presence of a maturase-like protein in the second intron in *psbC* of *Etl. gymnastica*^12^, which showed a weak similarity to *mat5* that is usually located in the *psbA* gene in Euglenales^19^, supports this scenario.

More surprisingly, another maturase gene, *mat2* was absent in two out of the three analyzed *Phacus* species (*P. inflexus* and *P. orbicularis*) and one *Lepocinclis* species (*L. tripteris*) (Fig. 2). Previously, it was shown to be missing in *P. orbicularis* which suggested the acquisition of *mat2* in the Euglenaceae lineage after the split from the Phacaceae^20^. Our results demonstrate that the majority of the Phacaceae possess *mat2*, which would imply the independent loss of that gene in some Phacaceae taxa.

It was proposed that proliferation of introns might be related to the number of maturases. Eutreptiales, with one maturase, has the lowest number of introns and Euglenacae with two or three maturases, tend to have more introns^20^. Our results confirmed the previously observed relation between the number of introns and number of maturases (Fig. 2). While *P. pleuronectes* was the only one of the Phacaceae that contained three maturases and had the highest number of introns (104) in this family. *Discoplastis* and all analyzed *Lepocinclis* strains contained two maturases and average (50–59) to a high (79–95) number of introns (Table 1) but never as high as *P. pleuronectes* (104 introns) (Table 1). Moreover, *P. inflexus* with one maturase contained only 29 introns, which is the lowest number of introns among Euglenales reported so far and only slightly higher number than in *Eutreptia viridis* (23 introns)^17^. The observation of the greater number of introns in strains with more maturases seems to be consistent but doesn’t explain the mechanism of intron proliferation.

An intron of *psbC*, which carries an intron-encoded maturase *ycf*13, is considered the ancestral intron because it is the only homologous intron in euglenophyte cpDNAs^46^. Surprisingly, intron1 in *psb*C of both strains of *L. tripteris* was not homologous with *E. gracilis psb*C intron2, though *L. tripteris psb*C intron1 still contained *ycf*13.

Two previous findings and conclusions about twintrons hold true with the newly analyzed genomes. We confirmed that twintrons present in *psbF* and *psbD* genes in *E. gracilis*^16^ are unique to that species and absent not only in all Euglenaceae^11,17,19,21,22^ but also in Phacaceae^20^ (Table 2). We also confirmed the presence of a twintron in *psbC* (intron1/Egra.2) (Table 2) in Phacaceae, which strongly supports the previously proposed ancestral origin of this intron^20^. Our data contradicted the suggestion of *petB* twintron (intron1) as a synapomorphy for all Euglenophyta^20,21^ as we identified the *petB* twintron in taxa representing all genera of Phacaceae except *L. ovum* and *P. pleuronectes* (Table 2). That might suggest that indeed the *petB* twintron is ancestral for the Euglenaceae but has been lost in at least two lineages of Phacaceae.

**Table 2:** Phacaceae pt genomes twintron analysis.

Twintron Site	*D. spathirhyncha*	*L. ovum*	*L. playfairiana*	*L. steinii*	*L. tripteris* (MI)	*L. tripteris* (UTEX)	*P. inflexus*	*P. pleuronectes*
atpE (intron1)	N	—	N	N	N	N	—	N
petB (intron1)	Y[26,4]	N	Y[9,1]	Y[19,2]	Y[3,1]	Y[3,1]	Y[10,2]	N
psbC (intron1/Eg.2/Ls.2/Pp.2)	Y	Y	Y	Y	NH	NH	NH	Y
psbC intron2/Eg.4/Ls.3/Lt.1/Pi.1/Pp.3)^a^	Y[3,1]	NH	Y	Y	Y	Y	Y[7,2]	Y
psbD (Eg.1)	NH	NH	NH	NH	NH	NH	NH	NH
psbD (Eg.8)	NH	NH	NH	NH	NH	NH	NH	NH
psbF (Eg.1)	—	—	—	—	—	—	—	—
psbK (intron2/Lo.1/Lt.1Pi.1/)	—	N	N	N	Y*	Y	N	N
psbT (intron1)	N	N	N	N	N	N	N	N
rpl16 (intron3/Pi.2)	N	N	N	N	N	N	N	N
rpoC1 (intron1)	N	N	N	N	N	N	N	N
rpoC1 (intron3)	NH	N	N	N	N	N	NH	N
rpoC1 (intron11/D.10/Lo.9/Ls.9/Pi.5)	N	NH	NH	N	NH	NH	N	NH
rps3 (intron1)	—	—	N	N	N	N	NH	N
rps18 (intron2)	Y*	N	N	N	N	N	N	Y[1,2]

Twintrons listed with common external intron followed by any deviation; number after the period corresponds to intron number for that taxon – D. = *D. spathirhyncha*, Eg. = *E. gracilis*, Lo. = *L. ovum*, Ls. = *L. steinii*, Lt. = L. tripteris, Pi. = *P. inflexus*, Pi. = *P. pleuronectes*. Group II twintrons in bold; all others are group III twintrons. N = No twintron found; NH = Non-Homologous external intron; Y = Potential twintron present [number of potential 5′ insertion sites, number of group III 3′ motifs]; —No intron in gene; *Putative twintron, <88 bp, ^a^suggested ancestral intron containing intron-encoded *ycf13*.

### Synteny, intrageneric and intraspecific variation

**Figure 3:**
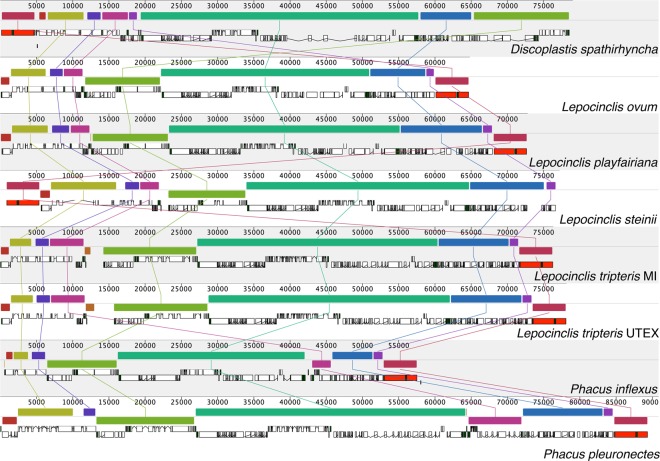
Synteny alignment of plastid genomes between Phacaceae representatives. Alignment performed in Mauve showing plastomes with one copy of the IR taken out. The order of plastomes, from top to bottom, is *D. spathirhyncha*, *L. ovum*, *L. playfairiana*, *L. steinii*, *L. tripteris* MI, *L. tripteris* UTEX, *P. inflexus*, and *P. pleuronectes*. Each colored block is a region of collinear sequence among all eight plastomes. Blocks on the top row are in the same orientation, while blocks on the bottom row are in inverse orientation. White and red boxes represent annotated CDS (protein-coding sequence) and rRNAs in the genomes respectively.

Synteny analyses revealed the same order of clusters within *Phacus* and *Lepocinclis* and some rearrangements among genera (Fig. 3). Those findings agreed with the observations within Euglenaceae. Taxa within a genus usually have the same arrangement of clusters and differences occur among genera^11,19,21,47^^.^

Intrageneric variability within the Euglenaceae was previously explored in *Euglena*^21,47^ and *Mononorphina*^11,21^, but not in Phacaceae. Newly sequenced pt genomes allowed to explore intrageneric variability within *Phacus* and *Lepocinclis*. In all previously analyzed cases, intrageneric evolution was limited, and significant changes occurred before the separation of those genera. Comparative studies of *Monomorphina* pt genomes revealed some differences, namely the presence of *mat5* and a higher number of introns in *M. parapyrum* resulting in the bigger size of the pt genome^11,21^. Finally, a comparison of the potential twintrons contained in the *Monomorphina* species revealed that the presence of twintrons was not conserved between taxa, suggesting that presence or absence of a twintron in a given intron is taxon-specific^21^. We observed similar differences within *Phacus* and *Lepocinclis*. In both genera, the number of introns varied greatly (50–95 for *Lepocinclis* and 29–104 for *Phacus*) (Table 1), and the main difference in the gene content is related to the presence/absence of maturases *mat5* and *mat2*. Analyses of twintrons (Table 2) also revealed some differences within genera and further supported earlier conclusions^21^ that twintrons are taxon-specific.

Plastid genomes of two strains of the *Lepocinclis tripteris* (MI 101 and UTEX 1311) were very similar, and ~90% of nucleotide sites were identical when compared across the entire genomes. This was a much higher identity than was observed in the two strains of *Euglena viridis*, which were only 70.5% identical^21^. The identity was even higher within coding space – 95.3%, which was also higher than between two *Euglena viridis* strains (91.5%). The pt genomes of the *L. tripteris* strains were also very similar in size but a comparison between the two genomes revealed that the area between tRNAs-S(GCU) and Y(GUA) were different (Fig. 1e,f): while both genomes contained two unidentified ORFs in this area, the two ORFs were different, and this region of the genome was 1,338 bp longer in UTEX 1311, which accounted for almost the entire difference in size between the genomes. Moreover, in both strains of *L. tripteris*, we observed the presence of a stop codon UAG in the 7^th^ amino acid of the *psbC* gene. Since *psb*C encodes a protein essential in photosynthesis, it is clear that this gene is still functional. Most likely the UAG codon is read-through^48^. Otherwise, it can be an example of RNA editing, observed so far in plastid transcripts of land plants and peridinin and fucoxanthin dinoflagellates^49,50^.

## Conclusions

Despite the fact that many Euglenaceae pt genomes have been characterized recently, surprisingly little was known about the plastid genomes of its sister family Phacaceae. To fill this gap, we have sequenced plastid genomes of eight taxa from all three genera classified in the Phacaceae. Gene content was highly conserved within the family, and the main differences were related to the presence/absence of maturases *mat2* and *mat5*. Genes were arranged into seven clusters, and their order was conserved within genera. No pattern of intron number was present in the Phacaceae, although we confirmed the correlation between the number of maturases and the number of introns. We also confirmed that twintrons present in *psbF* and *psbD* genes in *E. gracilis*^16^ are unique to that species and that the twintron in *psbC* is of ancestral origin. We rejected, however, the idea of the twintron in *petB* as a synapomorphy for all Euglenophyta, because it was not present in some Phacaceae.

Our study highlights the highly dynamic nature of the Euglenophyta plastid genomes, in particular with regards to the large IR sequence that experienced repeated losses, most probably at least once within the Phacaceae, once before the branching off of the Euglenaceae and once in the genus *Eutreptia*. It is necessary to analyze additional taxa from the basal lineages, such as Eutreptiales and *Rapaza*, to fully understand the dynamic history of the plastid genome in the Euglenophyta and decipher the mechanisms underlying the observed IR losses.

## Materials and Methods

### Culturing, sequencing, and annotation

The following eight cultures were used in this research: *Discoplastis spathirhyncha*, SAG 1224-42 (Experimental Phycology and Culture Collection of Algae at the University of Göttingen (EPSAG), Germany); *Lepocinclis ovum*, SAG 1244-8; *Lepocinclis playfairiana*, MI102 (strain isolated at Michigan State University); *Lepocinclis steinii*, UTEX 523 (The Culture Collection of Algae at The University of Texas at Austin, USA); *Lepocinclis tripteris*, UTEX 1331; *Lepocinclis tripteris*, (MI101); *Phacus inflexus*, ACOI 1336 (Coimbra Collection of Algae, Portugal); *Phacus pleuronectes* SAG 1261-3b. Cultures were maintained as described in^21^. *Lepocinclis playfairiana* and *L. tripteris* (MI) cells were identified in field samples collected from the East Lansing, MI, USA area and brought into culture in the following manner: single cells were picked from field samples using a sterile Pasteur pipette under a Leica MZ16 dissecting microscope (Leica Microsystems, Wetzlar, Germany) and transferred through a series of sterile drops of growth media, modified AF-6 medium^51^ with 150 mL L^−1^ of Soil-Water Medium (Carolina Biological Supply Company, Burlington, NC, USA), to ensure the presence of only one cell. The isolated cells were then placed into one well of a 96-well plate containing sterile growth media, one cell per well, and allowed to divide for ~2 weeks. Following this, well contents were moved into 12 mL culture tubes that contained sterile growth media and were allowed to grow for another ~2 weeks. Finally, the culture tubes were subsampled and viewed with a Zeiss Axioscope 2 plus microscope (Carl Zeiss Ing., Hallbergmoos, Germany) to ensure that they were uni-algal and verify the identity of the culture. These two cultures were also maintained as described previously^21^.

Cultures of *L. ovum*, *L. playfairiana*, *L. steinii*, *L. tripteris* (UTEX), *L. tripteris* (MI), *P. inflexus*, and *P. pleuronectes* were concentrated, washed, and had their DNA extracted with following protocols^52^, with the following alterations: Percoll (Research Organics, Cleveland, OH, USA) was substituted for Centricoll (Sigma Inc., St. Louis, MO, USA) in the *P. inflexus* process; *L. ovum*, *L. playfairiana*, *L. steinii*, and *P. pleuronectes* cultures were not run through the gradient step. DNA of *D. spathirhyncha* was extracted using phenol/chloroform, and DNA separation, using a cesium chloride gradient, was performed as described before^17^. All DNA was sequenced with Illumina paired-end reads at the Michigan State University Research Technology Support Facility: *D. spathirhyncha* with HiSeq 2 × 100 bp reads; all other taxa with MiSeq 2 × 150 bp reads. *Phacus inflexus* raw sequence data were assembled into contigs with the ‘De Novo Assemble…’ program in Geneious Pro version 6.8.1 (Biomatters Ltd, Auckland, New Zealand) as previously described^21^. All other raw sequence data were assembled into contigs with the ‘De Novo Assembly’ program in CLC Genomics Workbench version 5.5.1 (CLC Bio, Cambridge, Massachusetts, USA) as previously described^21^. The number of contigs and their lengths, number of reads per contig, and average coverage per contig are provided in the Supplementary Table 1.

All other aspects of genome discovery, including: identification of pt genome-containing contigs, joining of contigs (as necessary), sequence cleanup (as necessary), PCR primer creation, annotation, arrangement, and genome map creation were as performed as previously described^21^. The primers designed to confirm the presence of IRs are listed in the Supplementary Table S2. Additionally, when we were unable to identify the 5S in this pt genome through Rfam^53^, RNAmmer^54^, or by manual aligning, we used MFannot tool^55^ and confirmed its annotation by homology searches with 5S rRNA Database^56^. The search for potential twintrons was performed as previously described^21^, with the exception that instead of a manual search, a Python script was created (Supplementary File 1; the source code is available on GitHub https://github.com/ankarn/groupIII_twintrons) to find the 3′ conserved motif for Group III twintrons^9^ within the homologous external introns. Genome maps were drawn using OGDRAW^57^. Newly generated organelle genomes were deposited in GenBank (Table 1).

Synteny between the pt genomes of Phacaceae was determined and visualized with progressive Mauve 2.3.1^58^. Mauve performs syntenic comparisons between multiple genomes and displays these syntenic regions graphically. In the Mauve alignment the repeat regions of rRNA were not included because Mauve will not align repeat regions which have multiple matches in both genomes.

### Phylogenomic analyses

The 57 orthologous protein sequences and two rRNA nucleotide sequences encoded by 26 analyzed plastid genomes (Supplementary Table 3) were individually aligned with MAFFT ver. 7.271^59^, trimmed with BMGE ver. 1.12^60^, and concatenated with SequenceMatrix ver. 1.8^61^, leaving 11,499 reliably aligned amino acid (aa) positions and 3,959 reliably aligned nucleotide positions (rRNA). The protein data set was assembled from protein-coding genes: atpA, B, E, F, H, I, ccsA, petB, G, psaA, B, C, I, J, psbA, B, C, D, E, F, H, I, J, K, L, N, T, rbcL, rpl2, 5, 12, 14, 16, 20, 22, 23, 32, 36, rpoB, C1, C2, rps2, 3, 4, 7, 8, 9, 11, 12, 14, 18, 19, tufA, ycf4, 9, 12, 65. We excluded from the dataset sequences from the strains of the same species (*E. gracilis* var. *bacillaris* KP686076 and *E. viridis* KP686075). We excluded also *Colacium vesiculosum* (JN674636), which has been previously shown to have the unstable position at phylogenomic trees, and its elimination doesn’t lead to different topology but could reinforce the support for the relationships within the tree^33^. As an outgroup, we used three species representing Chlorophyta, previously shown to be the close relatives of Euglenophyta^33^. For Maximum Likelihood (ML) analyses each protein-coding gene was divided into a separate partition, and one rRNA partition was applied, resulting in 58 partitions. We determined the best choice of model for each partition with ModelFinder^62^ implemented in IQ-TREE ver. 1.6.1^63^. ML analyses were performed with IQ-TREE ver. 1.6.1^63^, using a partitioned analysis for multigene alignments under the recommended models^64^ and 1,000 ultrafast bootstrap^65^.

## Electronic supplementary material

Supplementary Information

## Data Availability

All the newly obtained sequences are deposited in GenBank under accession numbers MH898667-MH898674. All the sequence data set and analysis results obtained in this work are available from the corresponding author on reasonable request.
