# Supplementary material for: Dynamic evolution of inverted repeats in Euglenophyta plastid genomes
Source: Sci Rep. 2018 Oct 30;8:16071. doi: 10.1038/s41598-018-34457-w (PMC6207741; doi:10.1038/s41598-018-34457-w)
Supplement: Supplementary file 1 — Supplementary Information [file 41598_2018_34457_MOESM1_ESM.pdf]

Dynamic evolution of inverted repeats in Euglenophyta plastid genomes

Anna Karnkowska, Matthew S. Bennett, Richard E. Triemer

Supplementary Table S1. Assembly details for the taxa obtained in this study.

**Taxa**	**Assembly**	**Length**	**No. of reads**	**Average coverage depth**
Discoplastis spathirhyncha	Contig1	73,569 bp	2,018,259	2,521
	Contig2	5,211bp	259,034	4,365
Lepocinclis ovum	Contig1	60,008 bp	12,461	31
	Contig2	4,817 bp	1,660	51
L. playfairiana	Contig1	44,452bp	21,971	71
	Contig2	20,256bp	9,272	68
	Contig3	4,823bp	3,771	115
L. steinii	Contig1	71,359bp	24,497	51.6
	Contig2	5,204bp	2,724	77
L. tripteris (MI)	Contig1	70,204bp	66,184	145
	Contig2	6,623bp	5,722	129
	Contig3	1,700bp	200	18
L. tripteris (UTEX)	Contig1	80,808bp	133,412	258
Phacus inflexus	Contig1	55,874bp	125,849	338
P. pleuronectes	Contig1	92,122bp	81,375	134

Supplementary Table S2. The set of primers designed to confirm the presence of inverted repeats. The name of primers reflects the name of the gene they matched.

D. spathirhyncha
Q(UUG): TGATTTGGAGGTTCGAAT<br>23S: TGGATAACTGCTGAAAGCATA
23S: TGGATAACTGCTGAAAGCATA<br>chlI: GGACTTGAAAACATTTGA
L. ovum
23S: GTGGATAACTGCTGAAAGCATA<br>rps4: CTACCTCTATATCGTGACAT
23S: GTGGATAACTGCTGAAAGCATA<br>L(CAA): TCGTGGTGGAATGGTATACAC
L. playfairiana
rps4: CTACCTCTATAACGTGACAT<br>23S: GTGGATAACTGCTGAAGGCATA
23S: GTGGATAACTGCTGAAGGCATA<br>L(CAA): TCGTGGTGAAATGGTATACAC
L. steinii
rps4: CTACCTCGATAACGCGACAT<br>23S: GTGGATAACTGCTGAAGGCATA
23S: GTGGATAACTGCTGAAGGCATA<br>L(CAA): TCGTGGTGAAATGGTATACAC
***L. tripteris* (MI)**
23S: GTGGATAACTGCTGAAGGCAT<br>L(CAA): GCCTAAGGCTTGCCGGTTC
rps4: TTCTTAAACGTGGGCCTTTA<br>23S: GTGGATAACTGCTGAAGGCAT
***L. tripteris* (UTEX)**
rps4: TTCTTAAACGCGGGCCTTTA<br>23S: GTGGATAACTGCTGAAGGCAT
23S: GTGGATAACTGCTGAAGGCAT<br>L(CAA): GCCTAAGGCTTGCCGGTTC
P. inflexus
16S: AGCGTTCATCCTGAGCCAGGATCAA<br>L(CAA): TGAATCACGCATGTATACCA
P. pleuronectes
intergenic: ACTAGTCTTGCTTAGTTTTCAC<br>L(CAA): ACGTGTCTACCATTTCACCAT

Supplementary Table S3. Euglenophyta and Chlorophyta outgroup taxa used for phylogenomic analyses along with their accession numbers.

**Taxon name**	**Accession number**
Cryptoglena skujae	KP410781
Discoplastis spathirhyncha	MH898670
Euglena archaeoplastidiata	KP939040
Euglenaformis proxima	KC684276
Euglena gracilis	X70810
Euglena mutabilis	KT223519
Euglena viridis	JQ237893
Euglenaria anabaena	KP453743
Eutreptia viridis	JN643723
Eutreptiella gymnastica	NC_017754
Eutreptiella pomquetensis	KY706202
Lepocinclis ovum	MH898674
Lepocinclis playfairiana	MH898671
Lepocinclis steinii	MH898672
Lepocinclis tripteris (MI)	MH898668
Lepocinclis tripteris (UTEX)	MH898669
Monomorphina aenigmatica	JX457480
Monomorphina parapyrum	KP455987
Phacus inflexus	MH898667
Phacus orbicularis	KR921747
Phacus pleuronectes	MH898673
Strombomonas acuminata	JN674637
Trachelomonas volvocina	KP686077
Ostreococcus tauri	CR954199
Pycnococcus provasolii	FJ493498
Pyramimonas parkeae	FJ493499

Supplementary File 1. The custom python script to find 3’ motifs for group III twintrons.

```
##############################################################################
#
#               Search for Group III twinton 3' motifs
#
#   A program to find 3' motifs for group III twintrons,
#   given the external intron in FASTA # format.
#
# ** Program must be in same location as fasta file. **
#
# Assumption: only one sequence submitted in fasta file.
#
#   by: Matthew Bennett, Michigan State University
#
##############################################################################

# Function to complement a sequence
def complement(sequence):
    complement = ""
    for i in sequence:
        if i == "A":
            complement += "T"
        elif i == "T":
            complement += "A"
        elif i == "C":
            complement += "G"
        elif i == "G":
            complement += "C"

    return complement

matches = [] #Blank list for potential 3' matches.

while True:
    try:
        file_nm = input("FASTA file containg your external intron: ")
        file = open(file_nm, "r")
        break
    except FileNotFoundError:
        print ("\n", "FASTA file not found", "\n", sep = "")

#First line in FASTA is sequence name, strip of white space and get rid of ">"
seq_name = file.readline().strip()[1:]
# Second fasta line is sequence, strip of white space
seq = file.readline().strip()
```

```
# Strip the first 5 bases and last 5 bases, they only apply to external intron
seq = seq[5:-5]
# Reverse the sequence
rev_seq = seq[::-1]

# iterate through sequence and find an "A" to look for pattern:
#    abcdef (3–8 nucleotides) f'e'd' A c'b'a' (four nucleotides)
for i, base in enumerate(rev_seq):
    if base == "A":
        index = i
        search_seq_rev = rev_seq[(index-3):index] + rev_seq[(index+1):(index+4)]
        search_area_rev = rev_seq[(index+7):(index + 18)]
        search_seq_rc = complement(search_seq_rev)


        if len(search_seq_rev) == 6:
            search_area = search_area_rev[::-1]
            check = search_area.find(search_seq_rc)

            if check != -1:
                total_area_rev = rev_seq[(index-3):(index + 18)]
                total_area = total_area_rev[::-1]
                match_area = total_area[check:]
                match = (match_area)
                matches.append(match)

print ("\n", len(matches), " potential 3' motif(s) found in ",\
        file_nm, ":", "\n", sep = "")

for i in matches:
    print (i)

if len(matches) > 0:
    print ("\n", "*** Remember to add 4 bases to the end of any accepted\
 matching sequence ***", "\n", sep = "")
```
